# Traffic-related air toxics and preterm birth: a population-based case-control study in Los Angeles county, California

**DOI:** 10.1186/1476-069X-10-89

**Published:** 2011-10-07

**Authors:** Michelle Wilhelm, Jo Kay Ghosh, Jason Su, Myles Cockburn, Michael Jerrett, Beate Ritz

**Affiliations:** 1 Department of Epidemiology, School of Public Health, University of California, Los Angeles, USA; 2 Division of Environmental Health Sciences, School of Public Health, University of California, Berkeley, USA; 3 Department of Preventive Medicine, Keck School of Medicine, University of Southern California, USA

## Abstract

**Background:**

Numerous studies have associated air pollutant exposures with adverse birth outcomes, but there is still relatively little information to attribute effects to specific emission sources or air toxics. We used three exposure data sources to examine risks of preterm birth in Los Angeles women when exposed to high levels of traffic-related air pollutants - including specific toxics - during pregnancy.

**Methods:**

We identified births during 6/1/04-3/31/06 to women residing within five miles of a Southern California Air Quality Management District (SCAQMD) Multiple Air Toxics Exposure Study (MATES III) monitoring station. We identified preterm cases and, using a risk set approach, matched cases to controls based on gestational age at birth. Pregnancy period exposure averages were estimated for a number of air toxics including polycyclic aromatic hydrocarbons (PAHs), source-specific PM_2.5 _(fine particulates with aerodynamic diameter less than 2.5 μm) based on a Chemical Mass Balance model, criteria air pollutants based on government monitoring data, and land use regression (LUR) estimates of nitric oxide (NO), nitrogen dioxide (NO_2_) and nitrogen oxides (NO_x_). Associations between these metrics and odds of preterm birth were estimated using conditional logistic regression.

**Results:**

Odds of preterm birth increased 6-21% per inter-quartile range increase in entire pregnancy exposures to organic carbon (OC), elemental carbon (EC), benzene, and diesel, biomass burning and ammonium nitrate PM_2.5_, and 30% per inter-quartile increase in PAHs; these pollutants were positively correlated and clustered together in a factor analysis. Associations with LUR exposure metrics were weaker (3-4% per inter-quartile range increase).

**Conclusions:**

These latest analyses provide additional evidence of traffic-related air pollution's impact on preterm birth for women living in Southern California and indicate PAHs as a pollutant of concern that should be a focus of future studies. Other PAH sources besides traffic were also associated with higher odds of preterm birth, as was ammonium nitrate PM_2.5_, the latter suggesting potential importance of secondary pollutants. Future studies should focus on accurate modeling of both local and regional spatial and temporal distributions, and incorporation of source information.

## Background

Although numerous studies have associated air pollution exposure with risk of preterm and low weight birth, there is no consensus on pollutants or sources responsible for the biologic effects underpinning these findings. All of the routinely measured criteria pollutants (CO - carbon monoxide, NO_2 _- nitrogen dioxide, O_3 _- ozone, SO_2 _- sulfur dioxide, PM_10 _and PM_2.5 _- particulate matter with aerodynamic diameter less than 10 and 2.5 μm, respectively) have been linked to adverse birth outcomes across various pregnancy periods, some more consistently than others.

In our previous research in the Los Angeles (LA) Air Basin of Southern California, we reported most consistent associations between average levels of CO and particulate matter (measured as PM_10 _and PM_2.5_) during the first trimester and last six weeks prior to birth and risk of preterm birth [1-3]. Carbon monoxide is directly released as a combustion by-product and motor vehicles are a major source of this pollutant in the LA Basin [4]. PM_10 _and PM_2.5 _are less specific markers of traffic pollution since vehicle fuel combustion is just one source, along with industrial combustion emissions, secondary atmospheric reactions, biomass burning, meat cooking, paved road dust, and tire and brake wear debris [5,6]. Yet, our combined results for CO and PM suggest pollutants specific to traffic exhaust may be the causative agents of interest for preterm and low weight birth [7]. The potential importance of traffic pollutants to fetal development is further supported by our research associating residential proximity to traffic with risk of preterm birth [8], and residential levels of NO_2 _and PM_2.5 _estimated from air dispersion modeling of traffic emissions with risk of preeclampsia and preterm birth [9].

Although many investigators have linked average levels of CO and PM during pregnancy (among other criteria pollutants) and preterm birth [10-15], only four studies examined traffic effects using surrogate exposure measures based on traffic levels near homes [16-19], with two reporting null associations. A few studies have associated ambient and personal polycyclic aromatic hydrocarbon (PAH) levels during pregnancy with reduced fetal growth and preterm birth [20-23]. PAHs are of particular interest because they are fuel combustion by-products and can be carried in large quantities into the body by ultrafine particles (UFP, < 0.1 μm in aerodynamic diameter), the main size component of particulate matter directly released by on-road vehicles [24,25]. PAHs may disturb fetal development, possibly through adverse changes in placental transport or through oxidative stress pathways [26-28].

Since ambient air monitoring data are unlikely to adequately capture the greater spatial heterogeneity of pollutants directly emitted from traffic [29-31] and personal measurements of UFP and PAHs and other traffic exhaust constituents are too costly and logistically difficult in large population-based studies, investigators have used modeling techniques to estimate traffic exposures more accurately than simpler traffic proximity measures [32]. Land use regression (LUR) models based on pollution data collected via short-term intensive monitoring campaigns and Geographic Information Systems (GIS) information on pollution sources and meteorology are one example [33,34]. To date, six epidemiologic studies outside of the U.S. utilized LUR modeling techniques to examine traffic impacts on birth outcomes, with four studies examining preterm birth [35-38]. No associations were reported in two Dutch cohorts [36,37], while Llop et al. [35] reported increased risks of preterm birth with higher LUR-modeled exposure to NO_2 _and benzene in a Spanish cohort. Brauer et al. [38] reported stronger associations with very preterm birth (< 30 weeks gestation) for nitric oxide (NO) and PM_2.5 _exposures based on inverse-distance weighting of ambient monitoring data than with LUR estimates of NO and PM_2.5 _for women living in Vancouver.

Here we created pregnancy exposure estimates using three different data sources: (1) government monitoring data for criteria pollutants [39]; (2) LUR prediction surfaces for NO, NO_2 _and nitrogen oxides (NO_x_) [40] as more spatially-resolved traffic exposure models; and (3) a unique resource of air toxics monitoring data collected during 2004-2006 by the South Coast Air Quality Management District (SCAQMD) as part of the Multiple Air Toxics Exposure Study (MATES III). The latter data include information on atmospheric levels of a number of traffic-related air toxics, and in addition, provide estimates of source contributions to PM_2.5 _levels based on a Chemical Mass Balance (CMB) analysis. The goal of this study was to examine risk of preterm birth in Los Angeles women when exposed to high levels of traffic-related air pollutants prenatally using more spatially-resolved exposure models (LUR) as well as available data on specific toxics of biologic interest for this outcome (PAHs and PM_2.5 _from gasoline and diesel vehicles). We report results for a similar study of term low birth weight (LBW) separately [41].

## Methods

### Study Population

Electronic birth certificate records for all births occurring during 6/1/2004 to 3/30/2006 to women residing in LA County, California were assembled from the California Department of Health (n = 276,891). We excluded infants with recorded defects (n = 14,777), out-of-range gestational ages (missing, n = 12,159; < 140 days or > 320 days, n = 2,540), out-of-range birth weights (< 500 g or > 5000 g, n = 371), and non-singleton pregnancies (n = 5,629), leaving a total of 241,415 births. Although MATES air toxics measurements began on 4/1/2004, we selected births starting 6/1/2004 to ensure available monitoring data covered at least part of pregnancy (e.g., last 30 days of pregnancy).

For these 241,415 births, residential locations reported on birth certificates were mapped using a custom geocoder [42]. Of these, 110,429 (45.7%) were mapped to a specific parcel centroid, 47,181 (19.5%) using uniform lot interpolation, 69,421 (28.8%) using address range interpolation, and 13,587 (5.6%) based on zip code tabulation area centroid, city centroid, or county subdivision centroid; 797 non-geocodeable addresses were excluded. The geocoded residential locations were intersected with locations of seven MATES air toxics monitoring stations in LA County (Downtown Los Angeles, North Long Beach, Burbank, Pico Rivera, West Long Beach, Compton, and Huntington Park) and women living within five miles were selected (111,203 women, 46.2% of the 240,618). A radius of five miles was used to balance the need for a large sample size with an effort to reduce exposure misclassification assuming air pollution exposures for women living farther from a station are less well characterized.

From this cohort, we identified 10,265 preterm births (infants born < 37 completed weeks of gestation) and from among all infants still *in utero *at the gestational age when the preterm case was delivered, we randomly selected 10 eligible infants as matched controls (n = 102,650 controls). Among these 112,915 cases and controls, 4.1% were geocoded at the zip code centroid level (and none at a higher spatial level). Excluding these births did not change effect estimates reported below.

### Exposure Assessment

#### Monitoring Station Exposure Measures

For the air toxics of interest (see additional file 1: Table S-1), 24-hour averages collected every three days were extracted from MATES monitoring stations located within five miles of each woman's residence. As part of MATES III, monthly composite PM_2.5 _filter samples were speciated and the U.S. EPA CMB receptor model 8.2 was used to estimate diesel and other source contributions to PM_2.5 _levels in the basin during the two-year study period (see [43] for details). Monthly average PM_2.5 _concentrations (μg/m^3^) from the following sources were quantified based on the CMB model: diesel exhaust, gasoline exhaust, ammonium nitrate, ammonium sulfate, biomass burning, cooking operations, sea salt, geological (paved road dust), and residual oil burning.

We averaged these data over different pregnancy periods based on the birth date and gestational age reported on the birth certificate: first trimester (estimated first day of last menstrual period through day 92), second trimester (days 93-185), last 30 days of pregnancy, and the entire pregnancy. The lengths of second trimester and entire pregnancy averaging periods for controls were the same as for their matched case, i.e. these averaging periods for a preterm birth risk set were truncated on the gestational age of the case at birth. We implemented a 50% completeness criterion to ensure sufficient numbers of daily or monthly readings in each pregnancy averaging period (see additional file 1: Table S-2 for details).

For women residing within five miles of two or three stations, data from one station only were used to generate exposure averages if the other stations did not meet the completeness criteria. Otherwise, the 24-hour averages from two or three stations were weighted using the following equation:

P=∑i=1N(pidi)∑i=1N1di

Where P = weighted 24-hour average concentration of pollutant; p_*i *_= 24-hour average concentration of pollutant at station *i*; d_*i *_= distance to station *i*; and *N *is the number of monitoring stations within five miles. If none of the stations met the completeness criteria, the value for the exposure period was set to missing.

We also generated pregnancy exposure averages for criteria pollutants. Four of the seven MATES stations collected criteria pollutant data: Downtown LA, Burbank, North Long Beach and Pico Rivera (except PM_10_). Women residing near the three MATES stations without criteria pollutant data (West Long Beach, Compton and Huntington Park) were therefore linked to the four stations that did measure these pollutants, again using the 5 mile criterion. We also included criteria pollutant data from a fifth station, Lynwood, for women residing near the MATES Compton and Huntington Park stations since the five-mile buffers for these stations overlapped. No PM_10 _station was close enough to women residing within five miles of the Pico Rivera station, thus, these subjects are missing PM_10 _estimates. Hourly measurements for CO, NO_2_, NO, NO_x_, and ozone (O_3_) (10 am-6 pm) were first averaged over daily periods if sufficient data were available (see additional file 1: Table S-2). These daily averages for the gaseous pollutants and 24-hour measurements for PM_10 _and PM_2.5 _(collected every 6 and 3 days, respectively) were then averaged over pregnancy periods as described above, again implementing the completeness criteria in Table S-2. For subjects within five miles of two or three stations, daily values were weighted as described above.

#### Land Use Regression (LUR) Exposure Measures

As an alternative method to assess pregnancy exposures to traffic-related pollutants, we also extracted NO, NO_2_, and NO_x _concentration estimates at the residential locations from land use regression (LUR) model surfaces we developed for the LA Basin (see Su et al. [40] for details). The LUR surfaces were developed based on two-week average Ogawa NO_2 _and NO_x _measures we collected simultaneously in September 2006 and February 2007 at 181 locations throughout LA County. Final LUR models included the following variables: traffic counts, roadway lengths, distance to truck routes, land use characteristics, coordinates of the sampling sites, and satellite-derived soil brightness. The final regression models explained 81%, 86% and 85% of the variance in measured NO, NO_2 _and NO_x _concentrations, respectively, and cross validation analyses suggested a prediction accuracy of 87-91%.

The LUR models most closely approximate annual average concentrations; furthermore, the measurements used to calibrate the model were collected in 2006-2007. Thus, in addition to using the LUR annual average ("unseasonalized") estimates, we also created "seasonalized" LUR measures using government monitoring station measurements nearest to home locations to incorporate yearly and monthly air pollution variations. For example, the LUR estimates for NO were adjusted (multiplied) by the ratio of average ambient NO during each pregnancy month to annual average ambient NO (2006-2007) to generate pregnancy-month specific values. These seasonalized monthly LUR values were then averaged over each pregnancy period.

We applied the same hourly and daily exclusion criteria as described above when generating the pregnancy month scaling factors. The scaling factors for women within five miles of two or three stations were based on a weighted average, as described above for the criteria pollutant exposures.

### Statistical Analyses

To examine relationships among the various air pollution exposure variables, we calculated correlation coefficients and performed a factor analysis using principal components analysis for initial factor extraction and varimax rotation. The factor analysis was used to further examine clustering among the exposure measures and to determine whether the original variables could be represented more efficiently in statistical analyses of preterm birth by a smaller number of representative factors.

We estimated crude and adjusted effects of air pollution exposure on the odds of preterm birth using single- and multiple-variable conditional logistic regression models. We calculated odds ratios (ORs) and 95% confidence intervals (CIs) for both inter-quartile range (IQR) increases based on distributions in the entire dataset and specific unit-increases for each exposure metric. The IQR-based measures allow us to compare the size of effect estimates across different pollutants within the same pregnancy period, while the ORs for unit increases allow us to compare the size of estimates across different pregnancy periods for a given pollutant (note: since the distributions of pollutants are not the same across pregnancy periods, inter-quartile ranges differ accordingly).

We adjusted for maternal age, race/ethnicity, education, and parity as these variables were found to be important confounders in our previous analyses [1-3,7]. We also evaluated changes in odds ratio estimates when additionally controlling for (Table 1): prenatal care, payment source for prenatal care, whether the mother was born in the U.S., mother's birthplace, and a previously-developed socioeconomic status (SES) metric [44,45]. For the SES measure (standardized score for each census block group), principal component analysis was used to develop an index from seven U.S. Census 2000 variables. However, since these additional factors did not increase or decrease air pollution effect estimates by ≥ 5%, they were not included in final models.

**Table 1:** Demographic characteristics and crude odds ratios (95% CI) for preterm birth.^a^

Parameter	Preterm Cases (N = 10,265) n (%) or mean ± SD	Cohort (N = 111,203) n (%) or mean ± SD	Crude Preterm OR (95% CI)
**Gestational age (days)**	244 (17.0)	275 (14.6)	-- -- --

**Birth weight (g)**	2970 (643)	3342 (489)	--

**Infant's sex**			

Female	4790 (46.7)	54665 (49.2)	0.91 (0.88-0.95)

Male	5474 (53.3)	56537 (50.8)	1.00

Missing	1	1	

**Maternal age (years)**			

< 20	1336 (13.0)	13185 (11.9)	1.15 (1.08-1.24)

20-24	2555 (24.9)	28409 (25.6)	1.03 (0.97-1.09)

25-29	2556 (24.9)	29508 (26.5)	1.00

30-34	2121 (20.7)	24347 (21.9)	0.99 (0.94-1.06)

≥ 35	1697 (16.5)	15751 (14.2)	1.26 (1.18-1.35)

Missing		3	

**Maternal race/ethnicity**			

Hispanic	7749 (75.8)	83947 (75.7)	1.36 (1.25-1.47)

White, non-Hispanic	588 (5.8)	8696 (7.8)	1.00

African American	1014 (9.9)	8472 (7.6)	1.79 (1.62-1.98)

Asian	474 (4.6)	5938 (5.4)	1.19 (1.05-1.34)

Other^b^	398 (3.9)	3839 (3.5)	1.47 (1.29-1.66)

Missing	42	311	

**Maternal education (years)**			

≤ 8	1854 (18.2)	18169 (16.5)	1.09 (1.03-1.15)

9-12	5948 (58.6)	61957 (56.1)	1.00

13-15	1422 (14.0)	16605 (15.1)	0.88 (0.83-0.94)

≥ 16	935 (9.2)	13635 (12.3)	0.71 (0.66-0.77)

Missing	106	837	

**Parity**			

0	3535 (34.5)	41376 (37.2)	0.88 (0.84-0.92)

1 or more	6724 (65.5)	69787 (62.8)	1.00

Missing	6	40	

**Prenatal care**			

No prenatal care or started after 1st trimester	1287 (12.6)	10060 (9.1)	1.51 (1.41-1.60)

Started in first trimester	8935 (87.4)	100784 (90.9)	1.00

Missing	43	359	

**Mother's birthplace (U.S. vs outside U.S.)**			

U.S. Born	4096 (40.0)	42933 (38.6)	1.06 (1.01-1.10)

Foreign Born	6158 (60.0)	68187 (61.4)	1.00

Missing	11	83	

**Mother's birthplace**			

U.S.	4096 (40.0)	42933 (38.6)	1.00

Mexico	4048 (39.5)	45090 (40.6)	0.94 (0.90-0.98)

Other outside U.S. (includes Puerto Rico)	2110 (20.5)	23097 (20.8)	0.96 (0.91-1.01)

Missing	11	83	

**Primary payment for prenatal care**			

Private insurance/HMO/Pre-paid/Blue Cross-Blue Shield	2415 (23.9)	31685 (28.8)	1.00

Medi-Cal, other government programs, self pay, no care	7670 (76.1)	78283 (71.2)	1.30 (1.24-1.36)

Missing	180	1235	

**Census-based SES index (quintiles)**			

Q1	7237 (70.5)	74587 (67.1)	1.63 (1.32-2.02)

Q2	1710 (16.7)	19319 (17.4)	1.49 (1.20-1.85)

Q3	863 (8.4)	10737 (9.7)	1.35 (1.08-1.69)

Q4	364 (3.5)	5062 (4.5)	1.19 (0.94-1.50)

Q5	91 (0.89)	1498 (1.3)	1.00

^a ^Includes 10,265 preterm cases from the cohort of 111,203 births during June 1, 2004 to March 31, 2006 to women residing within five miles of a MATES air toxics monitoring station. Term LBW infants were eligible as preterm controls. Crude odds ratios are based on univariate conditional logistic regression analyses which take matching on gestational age into account.

^b ^Includes Native American/American Indian, Indian, Filipino, Hawaiian, Guamanian, Samoan, Eskimo, Aleut, Pacific Islander, Other (specified).

## Results

### Characteristics of Study Population

Women residing within five miles of MATES monitoring stations were younger, more likely to be Hispanic, more likely to be born in Mexico, less educated, and much more likely to use Medi-Cal or other government programs versus private insurance for prenatal care compared to the entire population of LA County mothers who delivered infants during the same time period (see additional file 1: Table S-3).

The prevalence of preterm birth in the study population was 9.2%. In univariate models, odds of preterm birth were greater for male infants, second or subsequent born infants, and infants born to younger (< 20 years) and older mothers (≥ 35 years) (Table 1). Higher odds of preterm birth were observed among infants born to non-White mothers, mothers receiving no prenatal care or initiating care after the first trimester, mothers utilizing Medi-Cal or other governmental programs to pay for prenatal care, and mothers with ≤ 8 years education. Infants of U.S.-born mothers had increased odds of preterm birth compared to those of foreign-born mothers, primarily driven by lower odds for infants of Mexican-born mothers. Odds of preterm birth were lower for infants born to mothers with ≥ 13 years of education compared to those with 9-12 years education.

### Exposure Metric Distributions and Correlations

#### Entire Pregnancy Averages

We provide information on distributions of entire pregnancy averages in Table 2, correlation coefficients in additional file 1: Table S-4, and factor analysis results in additional file 1: Table S-5. Extraction using principal components followed by varimax rotation suggested the 33 original entire pregnancy air pollution averages were best summarized by five factors. The first factor included monitoring-based averages for the following pollutants (Factor I): NO, NO_2 _and NO_x_, PM_2.5_, PM_10_, EC, OC, diesel PM_2.5_, total PAHs (naphthalene being the largest constituent by mass), benzene, biomass burning PM_2.5_, and ammonium nitrate; these pollutants tended to have higher concentrations in non-coastal areas. Factor II represented several pollutants with higher concentrations in coastal areas [43]: vanadium, residual oil PM_2.5_, and sea salt PM_2.5_. Vanadium and residual oil PM_2.5 _were strongly positively correlated, as this metal (along with nickel) was used to identify the residual oil combustion source in the CMB analysis. Other pollutants clustered within this "coastal" group were benzo(a)pyrene, gasoline PM_2.5_, and ozone (negatively). Benzo(g, h, i)perylene and CO averages loaded similarly on Factors I and II, indicating smaller differences between coastal and non-coastal concentrations. Ammonium sulfate PM_2.5 _loaded most strongly on a separate factor (Factor III), which was negatively related to most of the pollutants in Factor I, and positively related to most of the pollutants in Factor II. Concentrations of ammonium sulfate were higher along the coast, which could be due to aqueous-phase sulfate chemistry in higher relative humidity conditions [43]. However, its spatial patterns were different enough for it to be better represented by a factor separate from the "coastal" Factor II. Meat cooking PM_2.5 _loaded positively on Factor I and negatively on Factor III. The LUR metrics loaded separately on a fourth factor, reflecting their low correlation with the monitoring-based exposure metrics (additional file 1: Table S-4). Lastly, geological PM_2.5 _(modeled based on paved road dust samples using iron, calcium and silica as fitting species) was represented by a fifth factor. Interestingly, gasoline and diesel PM_2.5 _were not strongly spatially correlated (additional file 1: Table S-4) and loaded most strongly on separate factors. Although the factor analysis results helped identify and understand spatial patterns among the exposure measures, the five factors identified only explained 32% of the variance in the original variables. Thus, we elected to use the original variables in logistic regression models for preterm birth.

**Table 2:** Pollutant distributions for entire pregnancy averages.

	Pollutant^a^	n	Mean	IQR^b^	SD^c^
	NO	112915	27.3	7.5	7.5
	
**LUR_U^d^**	NO_2_	112915	25.2	4.2	3.6
	
	NO_x_	112915	52.8	11.6	10.5

	NO	86505	29.4	14.5	11.1
	
**LUR_S^e^**	NO_2_	86505	26.7	5.6	4.4
	
	NO_x_	86505	56.7	19.8	14.9

	Naphthalene	15006	181.7	52.2	34.6
	
	Benzo(a)pyrene	15006	0.13	0.06	0.05
	
	Benzo(g, h, i)perylene	15006	0.32	0.14	0.08
	
	Total PAHs	15006	221.4	59.3	38.6
	
	Benzene	61105	0.66	0.20	0.16
	
	TSP V	64224	10.5	5.1	5.6
	
	PM_2.5 _V	64095	7.5	3.5	4.1
	
	PM_10 _OC	64084	5.5	0.99	0.72
	
**Air toxics**	PM_10 _EC	64084	2.2	0.55	0.37
	
	PM_25 _OC	64055	7.4	1.3	0.95
	
	PM_25 _EC	64055	1.9	0.55	0.38
	
	Ammonium nitrate PM_2.5_	69936	6.2	1.8	1.1
	
	Ammonium sulfate PM_2.5_	69936	5.3	1.8	1.2
	
	Biomass burning PM_2.5_	69677	0.27	0.15	0.11
	
	Diesel PM_2.5_	69782	3.1	0.99	0.70
	
	Gasoline PM_2.5_	44691	1.3	0.71	0.44
	
	Geological PM_2.5_	66380	1.2	0.64	0.44
	
	Meat cooking PM_2.5_	55570	1.7	0.69	0.50
	
	Residual Oil PM_2.5_	69936	0.54	0.23	0.27
	
	Sea Salt PM_2.5_	69936	1.5	0.45	0.31

	CO	87815	0.84	0.38	0.27
	
	NO	87424	41.1	19.8	13.7
	
	NO_2_	87424	29.3	4.3	3.2
	
**Criteria**	NO_x_	87424	70.3	22.8	15.7
	
**pollutants**	O_3_	87815	34.5	8.2	5.8
	
	PM_10_	55687	31.4	5.8	3.7
	
	PM_2.5_	92865	18.0	2.6	2.1

^a ^Pollutant values are expressed in the following units: NO, NO_2_, NO_x_, O_3_, Benzene, ppb; B(a)P, PAHs, TSP V, PM_2.5 _V, ng/m^3^; PM_10_, PM_2.5_, PM_10 _OC, PM_10 _EC, PM_2.5 _OC, PM_2.5 _EC, μg/m^3^; CMB estimates for source contributions to PM_2.5_, μg/m^3^; CO, ppm.

^b ^Interquartile range.

^c ^Standard deviation.

^d ^Unseasonalized LUR model estimates.

^e ^Seasonalized LUR model estimates.

#### Trimester and Last Month of Pregnancy Averages

First trimester and last pregnancy month exposures for ambient measures of NO and NO_x _and total PAHs were strongly negatively correlated (r = -0.7). There were more moderate negative correlations between first trimester and last month averages (r = -0.5 to -0.6) for seasonalized LUR estimates of NO and NO_x_, benzene, EC and OC, and PM_2.5 _from diesel combustion and meat cooking. Correlations between first trimester and last month averages were lower for all other pollutants. Second trimester and entire pregnancy averages for all pollutants were strongly positively correlated (r = 0.7 to 0.9), except for B(a)P and sea salt PM_2.5 _(r = 0.6).

### Associations between Exposure Metrics and Preterm Birth

In models adjusted for maternal age, race/ethnicity, education, and parity, we estimated a 21% increase in odds of preterm birth per IQR increase in ammonium nitrate PM_2.5 _and 9-13% increases in odds for benzene, diesel PM_2.5_, PM_2.5 _OC and PM_2.5 _EC, and biomass burning PM_2.5 _(Table 3). Estimated odds increases were 14% and 6% for PM_10 _OC and PM_10 _EC, respectively. Inter-quartile range increases in entire pregnancy exposures to naphthalene, benzo(g, h, i)perylene and total PAHs were associated with approximately 30% odds increases and benzo(a)pyrene with a 13% odds increase, but these estimates relied on the smallest sample sizes since PAHs were measured at only two stations and for shorter time periods than the other pollutants.

**Table 3:** Associations between inter-quartile range increases in entire pregnancy average air pollution exposures and preterm birth based on single pollutant models.

Exposure Metric	Crude		Adjusted^a^	
	N (cases, controls)	OR (95% CI)	N (cases, controls)	OR (95% CI)
NO LUR_U^b^	10265, 102650	1.04 (1.02-1.06)	10134, 100467	1.03 (1.01-1.05)

NO LUR_S^c^	7838, 60115	1.01 (0.98-1.04)	7745, 58874	0.99 (0.96-1.02)

NO_2 _LUR_U	10265, 102650	1.04 (1.02-1.07)	10134, 100467	1.04 (1.02-1.07)

NO_2 _LUR_S	7838, 60115	0.96 (0.93-0.99)	7745, 58874	0.97 (0.94-1.00)

NO_x _LUR_U	10265, 102650	1.04 (1.02-1.06)	10134, 100467	1.03 (1.01-1.05)

NO_x _LUR_S	7838, 60115	0.99 (0.96-1.02)	7745, 58874	0.98 (0.95-1.01)

Naphthalene	1092, 1914	1.28 (1.14-1.44)	1068, 1859	1.29 (1.14-1.45)

Benzo(a)pyrene	1092, 1914	1.07 (0.97-1.18)	1068, 1859	1.13 (1.02-1.25)

Benzo(g, h, i)perylene	1092, 1914	1.28 (1.12-1.45)	1068, 1859	1.34 (1.17-1.52)

Total PAHs	1092, 1914	1.30 (1.15-1.46)	1068, 1859	1.30 (1.15-1.47)

Benzene	5856, 31564	1.10 (1.07-1.14)	5787, 30922	1.09 (1.06-1.13)

PM_10 _OC	6157, 34743	1.13 (1.09-1.17)	6083, 34040	1.14 (1.10-1.19)

PM_10 _EC	6157, 34743	1.05 (1.01-1.09)	6083, 34040	1.06 (1.01-1.10)

PM_25 _OC	6146, 34643	1.13 (1.09-1.17)	6072, 33940	1.13 (1.09-1.17)

PM_25 _EC	6146, 34643	1.13 (1.09-1.18)	6072, 33940	1.12 (1.08-1.17)

Ammonium nitrate PM_2.5_	6760, 41618	1.18 (1.13-1.24)	6673, 40737	1.21 (1.16-1.27)

Biomass burning PM_2.5_	6742, 41351	1.09 (1.05-1.13)	6655, 40476	1.12 (1.08-1.16)

Diesel PM_2.5_	6751, 41469	1.10 (1.06-1.14)	6664, 40592	1.11 (1.07-1.15)

^a ^Adjusted for maternal age, race/ethnicity and education, and parity.

^b ^Unseasonalized LUR model estimates.

^c ^Seasonalized LUR model estimates.

Negative associations were observed between preterm birth and entire pregnancy averages of vanadium, residual oil PM_2.5_, CO and PM_2.5 _(additional file 1: Table S-6). In multi-pollutant models including one representative pollutant from each of the five factors identified by the factor analysis, associations for vanadium and residual oil PM_2.5 _became slightly positive, but 95% CIs spanned the null value, while associations for CO and PM_2.5 _became positive with 95% CIs excluding the null (Table 4). Positive associations for the representative "non-coastal" pollutants, diesel and ammonium nitrate PM_2.5_, persisted in all multi-pollutant models.

**Table 4:** Associations between inter-quartile range increases in entire pregnancy average air pollution exposures and preterm birth based on multi-pollutant models.^a^

Pollutant	Model 1	Model 2	Model 3	Model 4	Model 5	Model 6	Model 7
NO LUR_S	1.01 (0.96-1.06)	1.03 (0.98-1.08)	1.03 (0.99-1.08)	1.03 (0.98-1.07)	1.02 (0.97-1.07)	1.02 (0.96-1.09)	1.03 (0.98-1.08)

Ammonium nitrate PM_2.5_	1.47 (1.34-1.62)	1.41 (1.29-1.53)	1.38 (1.29-1.48)	1.26 (1.15-1.38)	1.40 (1.31-1.50)	1.51 (1.39-1.64)	1.38 (1.29-1.47)

Ammonium sulfate PM_2.5_	0.87 (0.80-0.94)	0.90 (0.84-0.96)	0.91 (0.86-0.96)	0.91 (0.86-0.96)	0.93 (0.87-0.98)	0.83 (0.77-0.90)	0.90 (0.84-0.96)

Geological PM_2.5_	0.80 (0.75-0.85)	0.82 (0.77-0.87)	0.80 (0.76-0.85)	0.83 (0.78-0.88)	0.82 (0.77-0.87)	0.80 (0.74-0.87)	0.81 (0.77-0.86)

PM_2.5 _vanadium	1.02 (0.97-1.07)						

Residual oil PM_2.5_		1.02 (0.97-1.07)					

CO			1.04 (1.00-1.10)				

PM_2.5_				1.11 (1.02-1.20)			

O_3 _(10 am-6 pm)					0.96 (0.90-1.02)		

Gasoline PM_2.5_						0.95 (0.89-1.01)	

Sea salt PM_2.5_							1.02 (0.96-1.09)

^a ^All models adjusted for maternal age, race/ethnicity and education, and parity. Pollutants were selected to represent each of the five factors identified by the factor analysis. The same models, but replacing ammonium nitrate PM_2.5 _with diesel PM_2.5 _to represent Factor 1, indicated odds increases of 8-26% per IQR increase in entire pregnancy diesel PM_2.5_.

We estimated a 3-4% increase in odds of preterm birth per IQR increase in unseasonalized LUR measures of NO, NO_2 _and NO_x _(Table 3). Null associations between preterm birth odds and seasonalized LUR measures in single pollutant models became positive and similar in magnitude to effect estimates for the unseasonalized LUR measures in multi-pollutant models (Table 4).

Based on our analyses, no clear pattern emerged indicating pregnancy periods of greater susceptibility for preterm birth (results not shown). For seasonalized LUR measures and ambient measures of CO, NO, NO_2_, NO_x_, and PM_2.5_, the greatest positive associations were observed for last month of pregnancy averages. For PAHs, benzene, EC and OC, ammonium nitrate, and biomass burning and diesel PM_2.5_, positive associations were greatest for entire pregnancy and second trimester averages. Strong negative correlations between first trimester and last pregnancy month exposure averages for many pollutants, as well as strong positive correlations between second trimester and entire pregnancy averages, limit our ability to make conclusive statements regarding differences in risk across pregnancy periods.

## Discussion

We estimated 6-30% increases in odds of preterm birth per inter-quartile range increase in entire pregnancy exposures to OC, EC, benzene, PAHs, and diesel, biomass burning and ammonium nitrate PM_2.5_. These pollutants were positively correlated, underscored by their loading on a common factor, and had higher concentrations in winter than summer and inland compared to the coastal areas.

Our results for entire pregnancy averages appeared strongly driven by coastal versus non-coastal regional patterns in pollutant concentrations, with positive associations observed for pollutants with higher concentrations inland, and negative associations observed for pollutants with higher concentrations in coastal areas (vanadium, residual oil PM_2.5 _and CO). However, in multi-pollutant models (Table 4), positive associations for vanadium, residual oil PM_2.5 _and CO emerged, suggesting negative associations from single pollutant models reflected spatial (coastal versus non-coastal) and, to a lesser extent, temporal (winter versus summer) correlations. Positive associations we observed for the "non-coastal" pollutants in single pollutant models persisted in multi-pollutant models, indicating results were not purely driven by inland versus coastal comparisons, possibly due to risk factors for preterm birth other than air pollution that were not included in our analyses.

The negative associations estimated for geological and ammonium sulfate PM_2.5 _in multi-pollutant models may reflect the influence of meteorological factors with more favorable mixing and dispersion conditions for the other pollutants (e.g., levels of geological PM_2.5 _increase during higher wind conditions [43]) or may reflect negative correlations with pollutants like diesel PM_2.5 _and total PAHs that we were unable to adequately disentangle using this dataset and conventional logistic regression methods. Positive associations for entire pregnancy exposures to sea salt PM_2.5 _were closer to the null in multi-pollutant models.

We estimated 3-4% increases in odds of preterm birth per IQR increases in unseasonalized (annual average) LUR measures of NO, NO_2 _and NO_x_. Estimated associations between preterm birth odds and seasonalized LUR measures for the entire pregnancy period were null in single pollutant models, but became positive, and similar in magnitude to unseasonalized LUR measures, in multi-pollutant models. Associations between entire pregnancy averages of pollutants based on ambient monitoring data (PAHs, EC, OC, benzene, and diesel, biomass burning, and ammonium nitrate PM_2.5_) were greater in magnitude than those for the LUR exposure measures (based on IQR comparisons). This may reflect better representation of temporal and/or regional patterns in pollutant concentrations in the monitoring-based versus LUR measures. However, both types of metrics may be imperfect markers of the causal pollutants of interest. The LUR models were built on neighborhood-level NO, NO_2 _and NO_x _concentrations due to the relative ease of measurement with passive monitors deployed at many locations simultaneously; however, only two measurement periods were utilized to develop the models and it is still unclear how well NO, NO_2 _and NO_x _concentrations represent PAH concentrations at a local, neighborhood level. PAH concentrations have strong spatial and temporal variations in the LA Basin [46]. We used ambient monitoring station data to incorporate temporal variability due to meteorology into the seasonalized LUR measures. However, such temporal adjustment of LUR pollution surfaces may not be appropriate because of the un-validated assumption that ambient monitoring site measures and LUR modeled concentrations co-vary over space. Collection of a suite of air toxics including PAHs on a neighborhood scale and multiple times over a year, would help further examine the importance of local versus regional traffic pollutant exposure, but would be expensive and logistically difficult to implement.

Inter-quartile range increases in entire pregnancy PAH averages were associated most strongly with preterm birth risk. However, PAH data were only collected at two stations (West Long Beach and Downtown Los Angeles) from December 2004 through the end of March 2006. Compared to all women living within 5 miles of a MATES station, preterm cases and controls with entire pregnancy PAH averages available were slightly more likely to be foreign-born and to use government programs for health care (see additional file 1: Table S-3) and less likely to be in the highest SES quintiles, which may further limit generalizability of our PAH results.

We did not observe associations between entire pregnancy averages of NO, NO_2_, NO_x_, and PM_10 _based on government monitors and preterm birth, while in single pollutant models, CO and PM_2.5 _were negatively associated and O_3 _slightly positively associated with this outcome (additional file 1: Table S-6). However, in multi-pollutant models, associations for entire pregnancy CO and PM_2.5 _became positive, while the association for O_3 _became null. In pregnancy period analyses, positive associations were observed for last pregnancy month increases in CO, NO, NO_2_, NO_x _and PM_2.5_, suggesting the importance of temporal patterns in pollution concentrations for this outcome. These associations were observed despite the more limited spatial information available for the criteria pollutants (only 4 of 7 MATES monitors measured criteria pollutants and for the other three stations we relied on other, more distant stations within five miles). Despite differences in study areas, designs, populations, and time periods, these latest results for the criteria pollutants are similar to those reported in our previous studies. For example, in Wilhelm and Ritz [2] we reported odds ratio point estimates ranging between 1.01 and 1.08 per 1 ppm increase in CO (depending on pregnancy period and how close women lived to ambient monitoring stations), while here we estimated an OR of 1.04 per 0.38 ppm increase in entire pregnancy CO in multi-pollutant models. Wu et al. [9] reported an OR of 1.06 per 5.65 ppb increase in entire pregnancy NO_x _as estimated by an air dispersion model (CALINE) while here we estimated an OR of 1.03 per 11.5 ppb increase in unseasonalized LUR NO_x_. However, the CALINE model only included traffic emissions within 3000 m of women's residences while the LUR model included traffic parameters within 11,000 m and the two studies included very different geographical areas within the vast and complex LA metropolitan area (South LA/port areas and Orange County versus LA County coastal, urban core and eastern valley areas).

Oxidative stress caused by exposure to particles and associated toxics is one potential biological pathway of interest for air pollution's influence on preterm birth. Organic components of particulate matter, which comprise a large proportion of freshly emitted exhaust and secondary aerosols, can induce cytokine and chemokine expression in respiratory epithelium possibly due to cytotoxic reactive oxygen species (ROS) generated by PAHs, metals and related compounds; these inflammatory and oxidant stress responses are expected to occur at extrapulmonary sites as well [47,48]. Ultrafine particles in LA induced cellular heme oxygenase-1 expression and depleted intracellular glutathione, both important in oxidant stress responses, and were also shown to localize in mitochondria where they induce major structural damage which may also contributive to oxidative stress [49]. Cho et al. [50] reported the highest *in vitro *ROS formation in the UFP mode in LA Basin particles and a relatively high correlation of redox activity with elemental carbon (r^2 ^= 0.79), organic carbon (r^2 ^= 0.53) and benzo(g, h, i)perylene (r^2 ^= 0.82). Thus, a potential biologic mechanism through which UFPs and PAHs could exhibit their influence on adverse birth outcomes is through acting on oxidative stress and inflammatory pathways during pregnancy.

One limitation of this study was the relatively short time period (22 months) for which air toxics and speciated PM_2.5 _monitoring data were available. Because of seasonal fluctuations in air pollution concentrations within a given year (for example, ambient measures of NO and NO_x _and total PAHs exhibited strong seasonal variability with peaks in winter, while O_3 _and ammonium sulfate followed an opposite pattern with summer peaks) and because the number of births with available exposure measures was not equal across months in the study, there were moderate to strong negative correlations between first trimester and last pregnancy month exposure measures for many of the pollutants we evaluated. Second trimester exposure averages were highly positively correlated with entire pregnancy averages. These patterns limited our ability to identify pregnancy periods with greater susceptibility.

We used the SCAQMD's MATES III study results to estimate pregnancy period exposures to source-specific PM_2.5 _concentrations (diesel, gasoline, etc.). SCAQMD [43] provides a discussion of their data collection and source apportionment modeling methods. Because PM_2.5 _samples were composited for speciation analyses, only monthly-average source-specific PM_2.5 _values were available to derive pregnancy averages, thus temporal variation may not be well-represented. Here we used the CMB results based on the Northern Front Range Air Quality Study gasoline profile, as recommended by the SCAQMD, instead of CMB results based on the Department of Energy's Gasoline/Diesel Split Study gasoline profile [43]. Although we did not observe positive associations between entire pregnancy averages of gasoline PM_2.5 _and preterm birth, we estimated a 5% increase in odds per 0.5 μg/m^3 ^increase in second trimester exposure, similar to diesel PM_2.5 _effect estimates.

Because we relied on information recorded on California birth certificates for this study, we were unable to adjust for a number of potential confounding factors including active and passive smoking during pregnancy. In a previous population-based study incorporating survey data [3], we reported air pollution effect estimates for preterm birth adjusted for birth certificate variables (maternal age, education, race/ethnicity, and parity) did not change appreciably when we additionally adjusted for active or passive smoking or family income. Additionally, our population was predominately Hispanic (76%), with 68% of these mothers born outside the U.S. (54% in Mexico, 14% in other countries), and prenatal smoking rates among this group are low [51]. Our air pollution effect estimates did not change appreciably when we adjusted for prenatal care initiation, payment source for prenatal care, or for a Census-based measure of SES at the block group level.

For this study we used a risk set approach, matching preterm cases to controls based on gestational age at birth. This ensured each exposure period evaluated was the same length and covered the same developmental stages for cases and controls. Also, entire pregnancy averages for controls did not include exposures after 37 completed weeks of gestation when controls, by definition, are no longer at risk of becoming cases [14]. Matching on gestational age at birth versus birth date allowed us to maintain both spatial and temporal differences in air pollution exposures, a strength of our study.

A major strength of this study was the use of novel air pollution exposure information in addition to routine, government monitoring station data for criteria pollutants, which has so far been the predominant method of exposure assessment in birth outcome studies. By using PAH monitoring data in concert with source-specific PM_2.5 _information from a CMB model, we detected positive associations between prenatal exposure to PAHs and odds of preterm birth and our results suggest PM_2.5 _from diesel combustion may be a particularly important exposure source, but not necessarily the only exposure source of interest (we also observed associations with biomass burning PM_2.5_, and to a lesser extent, meat cooking PM_2.5_, other PAH sources). Associations with ammonium nitrate PM_2.5 _suggest secondary pollutant formation through atmospheric reactions may be important for this outcome. Altogether, the air pollution exposure measures used here allowed us to better pinpoint sources and pollutants (PAHs) as targets of future analyses. Additional studies utilizing air toxics in addition to criteria pollutant data and source-specific information on pollutant contributions would provide further evidence on how and which air pollutants impact fetal development and may help inform regulatory policy decisions.

## Conclusions

Using three different exposure information sources, this analysis provides additional evidence for the impact of traffic-related air pollution on preterm birth in women living in Southern California. Our results point to PAHs as pollutants of special concern that should be a focus of future studies. PAH sources other than traffic also contributed to higher odds of preterm birth, as did ammonium nitrate PM_2.5_, the latter suggesting a role for secondary pollutants. Future studies should focus on accurate modeling of both local and regional spatial and temporal air pollution distributions, and incorporate source information to help better inform policy decisions on air pollution control.

## List of Abbreviations

CI: confidence interval; CMB: Chemical Mass Balance; CO: carbon monoxide; EC: elemental carbon; GIS: Geographic Information System; IQR: inter-quartile range; LA: Los Angeles; LBW: low birth weight; LUR: land use regression; MATES: Multiple Air Toxics Exposure Study; NO: nitric oxide; NO_2_: nitrogen dioxide; NO_x_: nitrogen oxides; O_3_: ozone; OC: organic carbon; PAHs: polycyclic aromatic hydrocarbons; PM_2.5_: particulate matter < 2.5 μm in aerodynamic diameter; PM_10_: particulate matter < 10 μm in aerodynamic diameter; ROS: reactive oxygen species; SCAQMD: South Coast Air Quality Management District; SES: socioeconomic status; SO_2_: sulfur dioxide; UFP: ultrafine particles; particles < 0.1 μm in aerodynamic diameter; U.S. EPA: United States Environmental Protection Agency.

## Competing interests

The authors declare that they have no competing interests.

## Authors' contributions

MW, BR and MJ designed the original study, and with JKG, JS and MC, analyzed and interpreted the data, and wrote the manuscript. All authors read and approved the final manuscript.

## Supplementary Material

Additional file 1 **Supplementary Material, Tables S1 through S6**. Click here for file
