# Supplementary material for: Traffic-related air toxics and preterm birth: a population-based case-control study in Los Angeles county, California
Source: Environ Health. 2011 Oct 7;10:89. doi: 10.1186/1476-069X-10-89 (PMC3204282; doi:10.1186/1476-069X-10-89)
Supplement: Additional file 1 — Supplementary Material, Tables S1 through S6. [file 1476-069X-10-89-S1.DOC]

# Additional file 1

# Traffic-related air toxics and preterm birth: a population-based case-control study in Los Angeles County, California

Michelle Wilhelm^1^, Jo Kay Ghosh^1^, Jason Su^2^, Myles Cockburn^3^, Michael Jerrett^2^, Beate Ritz^1^

^1^ Department of Epidemiology, School of Public Health, University of California, Los Angeles
^2^ Division of Environmental Health Sciences, School of Public Health, University of California, Berkeley

^3^ Department of Preventive Medicine, Keck School of Medicine, University of Southern California

## Table S-1. List of air toxics included in study.

| Total PAHs:  naphthalene, benzo(a)pyrene, benzo(a)anthracene, benzo(b+j+k)fluoranthene, benzo(g,h,i)perylene, indeno(1,2,3-cd)pyrene, dibenzo(a,h)anthracene, chrysene, anthracene, pyrene, fluoranthene, acenapthylene, acenaphthene, fluorene, and phenanthrene |
| --- |
| Naphthalene^a^ |
| Benzo(a)pyrene |
| Benzo(g,h,i)perylene |
| Benzene^b^ |
| Toluene^a^ |
| Ethyl benzene |
| m+p-Xylenes |
| o-Xylenes |
| Vanadium^c^ |

(a) We evaluated naphthalene separately because its levels were on average much higher than those of other PAHs (SCAQMD, 2008), benzo(a)pyrene separately due to reported health effects in previous studies (Perera et al. 2004) and benzo(g,h,i)perylene separately because of its relatively high correlation with UFP redox activity (Cho et al. 2005).

(b) Benzene, toluene, ethyl benzene and xylenes were selected as VOCs indicative of traffic exhaust (Beckerman et al. 2008). Because all four compounds were highly correlated and produced similar results, we present results for benzene only.

(c) We evaluated the metal vanadium due to the importance of port emissions in the LA Basin (vanadium was used as a marker of residual oil combustion by ships by SCAQMD).

## Table S-2. Exclusion criteria used when estimating pregnancy period specific air pollution exposure metrics.

| **Pollutant** | **Criteria** |
| --- | --- |
| MATES air toxics  PAHs^a^, BTEX, Vanadium, OC and EC^b^ | We required at least 5 available readings for each full pregnancy month in a given period (trimester or entire pregnancy) as well as 5 readings during the last 30 days of a given period. |
| MATES CMB PM_2.5_ concentrations^b,c^ | The source-specific PM_2.5_ concentration estimates from the CMB analysis were available on a monthly basis (because PM_2.5_ samples had to be composited for speciation). Thus, we took weighted averages of calendar month-specific concentration values based on when pregnancy months occurred. For example, for a woman with a first pregnancy month from January 17 to February 15 (we assumed a 30-day month to simplify calculations), we weighted the corresponding January and February CMB concentration estimates by 50%.  We also calculated weighted averages of the number of calendar month readings available for each full pregnancy month. For example, for a woman with a first pregnancy month from January 17 to February 15 and with a CMB estimate available for both months, the weighted average number of readings was one. For each full pregnancy month in a given trimester, we required at least 75% of the pregnancy month to fall in calendar month(s) with a CMB source-specific PM_2.5_ value available (i.e., weighted average number of readings >0.75). We also required at least 75% of the last 30 days of pregnancy to fall in calendar month(s) with a CMB reading available.  We relaxed this criterion for the entire pregnancy period averages since ~15% of monthly values were missing for the gasoline and meat cooking source categories. For entire pregnancy averages, we required at least 75% of all full months during pregnancy to have a CMB value available. For example, for a woman who completed 8 pregnancy months, we required at least 6 monthly CMB readings to be available (based on the sum of the weighted number of readings). We also required at least 50% of the last 30 days of pregnancy to fall in calendar month(s) with a CMB reading available. |
| CO | Criteria for hourly measurements:  At least 50% of hourly values available per 24-hr period and at least 50% of hourly values available from 6am-6pm. If sufficient data were available, a daily (24-hour) average was generated based on the hourly data.  Criteria for pregnancy periods:  We required at least 15 readings for each full month in a given period (trimester or entire pregnancy) as well as 15 readings during the last 30 days of the pregnancy period. |
| NO, NO_2_, NO_x_ | Criteria for hourly measurements:  At least 50% of hourly values available per 24-hr period and at least 50% of hourly values available from 8am-8pm.  Criteria for pregnancy periods:  We required at least 15 readings for each full month in a given period (trimester or entire pregnancy) as well as 15 readings during the last 30 days of the pregnancy period. |
| O_3_ (10am-6pm) | Criteria for hourly measurements:  At least 50% of hourly values available from 10am-6pm.  Criteria for pregnancy periods:  We required at least 15 readings for each full month in a given period (trimester or entire pregnancy) as well as 15 readings during the last 30 days of the pregnancy period. |
| PM_10_ | We required 3 or more values to be available per each full pregnancy month and during the last 30 days of pregnancy. |
| PM_2.5_^c^ | We required 5 or more values to be available per each full pregnancy month and during the last 30 days of pregnancy. |

(a) PAH data were only available at the Downtown Los Angeles and West Long Beach stations from 12/8/04-3/30/06. Collection of PAHs was reduced from every 3 days to every 6 days during May-September. Since there were few missing values in the PAH data during the 3 day schedule, we based the exclusion criteria on the 6 day schedule, i.e., required at least 3 readings for each full month of pregnancy as well as during the last 30 days of pregnancy for each period evaluated. (b) At the Huntington Park station, data were collected from April 2004 through May 2005 only. At Pico Rivera, data were available from April 2004 through May 2005 and August 2005 through March 2006, except source-specific PM_2.5_ concentrations from the CMB model were only available during April 2004 through May 2005. (c) PM_2.5_ measures were available daily at the North Long Beach and Downtown Los Angeles stations.

## Table S-3. Demographic characteristics (n, %) of cohort used to select preterm case-control sample (n=111,203) compared to all births in LA County during 6/1/2004 to 3/31/2006.

| **Parameter** | **All LA County births (N=241,415)** | **Geocoded births**  **(N=240,618)** | **Geocoded births within 5 mi of a MATES station**  **(N=111,203)** | **Preterm cases and controls with entire pregnancy PAH values**  **(n=15,006)** |
| --- | --- | --- | --- | --- |
| **Gestational age (days) (mean, SD)** | 275 (14.3) | 275 (14.3) | 275 (14.6) | 273 (15.3) |
| **Birth weight (g) (mean, SD)** | 3348 (484) | 3348 (484) | 3342 (489) | 3308 (498) |
| **Infant’s sex** |  |  |  |  |
| Female | 118471 (49.1) | 118088 (49.1) | 54665 (49.2) | 7238 (48.2) |
| Male | 122943 (50.9) | 122529 (50.9) | 56537 (50.8) | 7768 (51.8) |
| Missing | 1 | 1 | 1 |  |
| **Maternal age (years)** |  |  |  |  |
| <20 | 23148 (9.6) | 23076 (9.6) | 13185 (11.9) | 1812 (12.1) |
| 20-24 | 53660 (22.2) | 53480 (22.2) | 28409 (25.6) | 3796 (25.3) |
| 25-29 | 62374 (25.8) | 62156 (25.8) | 29508 (26.5) | 3927 (26.2) |
| 30-34 | 59535 (24.7) | 59357 (24.7) | 24347 (21.9) | 3224 (21.4) |
| ≥35 | 42695 (17.7) | 42546 (17.7) | 15751 (14.2) | 2247 (15.0) |
| Missing | 3 | 3 | 3 |  |
| **Maternal race/ethnicity** |  |  |  |  |
| Hispanic | 151559 (63.1) | 151146 (63.1) | 83947 (75.7) | 11326 (75.7) |
| White, non-Hispanic | 41734 (17.4) | 41501 (17.3) | 8696 (7.8) | 968 (6.5) |
| African American | 18262 (7.6) | 18210 (7.6) | 8472 (7.6) | 857 (5.7) |
| Asian | 18163 (7.6) | 18121 (7.6) | 5938 (5.4) | 1013 (6.8) |
| Other^a^ | 10578 (4.4) | 10536 (4.4) | 3839 (3.5) | 793 (5.3) |
| Missing | 1119 | 1104 | 311 | 49 |
| **Maternal education (years)** |  |  |  |  |
| ≤8 | 30319 (12.7) | 30228 (12.7) | 18169 (16.5) | 2651 (17.9) |
| 9-12 | 115264 (48.3) | 114917 (48.3) | 61957 (56.1) | 8253 (55.6) |
| 13-15 | 41126 (17.2) | 40961 (17.2) | 16605 (15.1) | 2071 (14.0) |
| ≥16 | 51933 (21.8) | 51764 (21.8) | 13635 (12.3) | 1862 (12.5) |
| Missing | 2773 | 2748 | 837 | 169 |
| **Parity** |  |  |  |  |
| 0 | 94981 (39.3) | 94623 (39.3) | 41376 (37.2) | 5724 (38.2) |
| 1 or more | 146368 (60.7) | 145931 (60.7) | 69787 (62.8) | 9281 (61.8) |
| Missing | 66 | 64 | 40 | 1 |
| **Prenatal care** |  |  |  |  |
| No care or after 1st trimester | 20937 (8.7) | 20822 (8.7) | 10060 (9.1) | 1261 (8.4) |
| Started in first trimester | 219865 (91.3) | 219186 (91.3) | 100784 (90.9) | 13740 (91.6) |
| Missing | 613 | 610 | 359 | 5 |
| **Mother’s birthplace (US vs outside US)** |  |  |  |  |
| U.S. Born | 106083 (44.0) | 105685 (44.0) | 42933 (38.6) | 5159 (34.4) |
| Foreign Born | 135082 (56.0) | 134685 (56.0) | 68187 (61.4) | 9829 (65.9) |
| Missing | 250 | 248 | 83 | 18 |
| **Mother’s birthplace** |  |  |  |  |
| U.S. | 106083 (44.0) | 105685 (44.0) | 42933 (38.6) | 5159 (34.4) |
| Mexico | 77685 (32.2) | 77488 (32.2) | 45090 (40.6) | 6066 (40.5) |
| Other outside U.S. (includes Puerto Rico) | 57397 (23.8) | 57197 (23.8) | 23097 (20.8) | 3763 (25.1) |
| Missing | 250 | 248 | 83 | 18 |
| **Primary payment for prenatal care** |  |  |  |  |
| Private insurance/HMO/Pre-paid/Blue Cross-Blue Shield | 98299 (41.6) | 97971 (41.6) | 31685 (28.8) | 3735 (25.2) |
| Medi-Cal, other government programs, self pay, no care | 137760 (58.4) | 137324 (58.4) | 78283 (71.2) | 11110 (74.8) |
| Missing | 5356 | 5323 | 1235 | 161 |

(a) Includes Native American/American Indian, Indian, Filipino, Hawaiian, Guamanian, Samoan, Eskimo, Aleut, Pacific Islander, Other (specified).

## Table S-4. Pearson’s correlation coefficients for entire pregnancy averages.

|  |  | **Pearson Correlation Coefficients** | | | | | | | | | | | | | | | | | | | | | | | | | | | | | | |
| --- | --- | --- | --- | --- | --- | --- | --- | --- | --- | --- | --- | --- | --- | --- | --- | --- | --- | --- | --- | --- | --- | --- | --- | --- | --- | --- | --- | --- | --- | --- | --- | --- |
|  |  | **LUR_U** | | | **LUR_S** | | | **Air toxics** | | | | | | | | | | | | | | | | | | | **Criteria Pollutants** | | | | | |
|  |  | NO | NO_2_ | NO_x_ | NO | NO_2_ | NO_x_ | NAPH | BAP | BGP | PAHS | BENZ | PM_2.5_ V | PM_10_ OC | PM_10_ EC | PM_25_ OC | PM_25_ EC | AN | AS | BB | D | G | GL | MC | RO | SS | CO | NO | NO_2_ | O_3_ | PM_10_ | PM_2.5_ |
| **LUR_U^a^** | NO | 1.0 |  |  |  |  |  |  |  |  |  |  |  |  |  |  |  |  |  |  |  |  |  |  |  |  |  |  |  |  |  |  |
|  | NO_2_ | 0.80 | 1.0 |  |  |  |  |  |  |  |  |  |  |  |  |  |  |  |  |  |  |  |  |  |  |  |  |  |  |  |  |  |
|  | NO_x_ | 0.94 | 0.90 | 1.0 |  |  |  |  |  |  |  |  |  |  |  |  |  |  |  |  |  |  |  |  |  |  |  |  |  |  |  |  |
| **LUR_S^b^** | NO | 0.65 | 0.47 | 0.61 | 1.0 |  |  |  |  |  |  |  |  |  |  |  |  |  |  |  |  |  |  |  |  |  |  |  |  |  |  |  |
|  | NO_2_ | 0.52 | 0.62 | 0.59 | 0.63 | 1.0 |  |  |  |  |  |  |  |  |  |  |  |  |  |  |  |  |  |  |  |  |  |  |  |  |  |  |
|  | NO_x_ | 0.60 | 0.51 | 0.64 | 0.95 | 0.78 | 1.0 |  |  |  |  |  |  |  |  |  |  |  |  |  |  |  |  |  |  |  |  |  |  |  |  |  |
| **Air toxics** | NAPH^c^ | 0.14 | 0.27 | 0.16 | 0.56 | 0.38 | 0.51 | 1.0 |  |  |  |  |  |  |  |  |  |  |  |  |  |  |  |  |  |  |  |  |  |  |  |  |
|  | BAP | -0.23 | -0.42 | -0.26 | 0.44 | 0.25 | 0.48 | 0.44 | 1.0 |  |  |  |  |  |  |  |  |  |  |  |  |  |  |  |  |  |  |  |  |  |  |  |
|  | BGP | -0.07 | -0.12 | -0.08 | 0.59 | 0.37 | 0.59 | 0.82 | 0.87 | 1.0 |  |  |  |  |  |  |  |  |  |  |  |  |  |  |  |  |  |  |  |  |  |  |
|  | PAHS | 0.12 | 0.25 | 0.15 | 0.57 | 0.39 | 0.53 | 1.0 | 0.46 | 0.83 | 1.0 |  |  |  |  |  |  |  |  |  |  |  |  |  |  |  |  |  |  |  |  |  |
|  | BENZ | -0.02 | -0.05 | -0.02 | 0.36 | 0.11 | 0.33 | 0.90 | 0.25 | 0.65 | 0.89 | 1.0 |  |  |  |  |  |  |  |  |  |  |  |  |  |  |  |  |  |  |  |  |
|  | PM_2.5_ V^d^ | -0.18 | -0.47 | -0.22 | -0.06 | -0.07 | 0.00 | -0.38 | 0.57 | 0.16 | -0.33 | -0.31 | 1.0 |  |  |  |  |  |  |  |  |  |  |  |  |  |  |  |  |  |  |  |
|  | PM_10_ OC | -0.04 | 0.02 | -0.04 | 0.38 | 0.23 | 0.36 | 0.85 | 0.31 | 0.66 | 0.87 | 0.76 | -0.35 | 1.0 |  |  |  |  |  |  |  |  |  |  |  |  |  |  |  |  |  |  |
|  | PM_10_ EC | -0.02 | -0.02 | -0.02 | 0.48 | 0.31 | 0.48 | 0.78 | 0.63 | 0.83 | 0.79 | 0.59 | -0.19 | 0.82 | 1.0 |  |  |  |  |  |  |  |  |  |  |  |  |  |  |  |  |  |
|  | PM_25_ OC | 0.10 | 0.20 | 0.12 | 0.34 | 0.18 | 0.30 | 0.66 | -0.16 | 0.26 | 0.66 | 0.64 | -0.53 | 0.86 | 0.76 | 1.0 |  |  |  |  |  |  |  |  |  |  |  |  |  |  |  |  |
|  | PM_25_ EC | 0.06 | 0.05 | 0.06 | 0.52 | 0.31 | 0.51 | 0.79 | 0.40 | 0.69 | 0.82 | 0.62 | -0.19 | 0.86 | 0.91 | 0.86 | 1.0 |  |  |  |  |  |  |  |  |  |  |  |  |  |  |  |
|  | AN | 0.15 | 0.39 | 0.18 | 0.15 | 0.31 | 0.15 | 0.64 | -0.30 | 0.16 | 0.64 | 0.25 | -0.64 | 0.58 | 0.39 | 0.67 | 0.47 | 1.0 |  |  |  |  |  |  |  |  |  |  |  |  |  |  |
|  | AS | -0.01 | -0.10 | -0.02 | -0.42 | -0.09 | -0.35 | -0.61 | -0.04 | -0.36 | -0.54 | -0.40 | 0.43 | -0.32 | -0.43 | -0.30 | -0.32 | 0.09 | 1.0 |  |  |  |  |  |  |  |  |  |  |  |  |  |
|  | BB | -0.08 | -0.04 | -0.09 | 0.38 | 0.22 | 0.37 | 0.69 | 0.55 | 0.74 | 0.70 | 0.05 | 0.12 | 0.31 | 0.32 | 0.09 | 0.35 | 0.05 | -0.50 | 1.0 |  |  |  |  |  |  |  |  |  |  |  |  |
|  | D | 0.09 | 0.20 | 0.10 | 0.44 | 0.19 | 0.39 | 0.70 | -0.03 | 0.37 | 0.72 | 0.40 | -0.36 | 0.75 | 0.68 | 0.84 | 0.84 | 0.47 | -0.56 | 0.54 | 1.0 |  |  |  |  |  |  |  |  |  |  |  |
|  | G | -0.05 | -0.22 | -0.07 | 0.31 | 0.00 | 0.29 | 0.21 | 0.80 | 0.61 | 0.26 | 0.65 | 0.08 | 0.57 | 0.55 | 0.51 | 0.60 | 0.04 | -0.16 | -0.13 | 0.34 | 1.0 |  |  |  |  |  |  |  |  |  |  |
|  | GL | 0.05 | 0.25 | 0.07 | 0.24 | 0.21 | 0.21 | 0.57 | -0.10 | 0.24 | 0.53 | 0.41 | -0.66 | 0.62 | 0.50 | 0.61 | 0.45 | 0.58 | -0.40 | 0.06 | 0.50 | 0.29 | 1.0 |  |  |  |  |  |  |  |  |  |
|  | MC | 0.02 | -0.07 | 0.01 | 0.42 | 0.18 | 0.40 | 0.80 | 0.27 | 0.60 | 0.77 | 0.92 | -0.16 | 0.63 | 0.59 | 0.55 | 0.62 | 0.20 | -0.27 | -0.09 | 0.32 | 0.70 | 0.32 | 1.0 |  |  |  |  |  |  |  |  |
|  | RO | -0.17 | -0.47 | -0.21 | -0.06 | -0.07 | 0.00 | -0.39 | 0.57 | 0.15 | -0.34 | -0.30 | 0.99 | -0.31 | -0.12 | -0.49 | -0.12 | -0.64 | 0.38 | 0.12 | -0.33 | 0.09 | -0.67 | -0.15 | 1.0 |  |  |  |  |  |  |  |
|  | SS | -0.02 | -0.14 | -0.02 | -0.06 | 0.15 | 0.01 | -0.02 | 0.82 | 0.53 | 0.02 | 0.16 | 0.34 | 0.04 | 0.08 | -0.12 | 0.06 | 0.15 | 0.69 | -0.30 | -0.48 | 0.14 | -0.29 | 0.38 | 0.32 | 1.0 |  |  |  |  |  |  |
| **Criteria Pollutants** | CO | 0.01 | -0.10 | -0.01 | 0.32 | -0.11 | 0.25 | 0.55 | 0.72 | 0.78 | 0.61 | 0.83 | -0.19 | 0.54 | 0.36 | 0.47 | 0.45 | 0.12 | -0.17 | -0.13 | 0.22 | 0.73 | 0.26 | 0.75 | -0.21 | 0.12 | 1.0 |  |  |  |  |  |
|  | NO^e^ | 0.03 | 0.01 | 0.03 | 0.60 | 0.13 | 0.54 | 0.92 | 0.53 | 0.85 | 0.92 | 0.87 | -0.29 | 0.75 | 0.71 | 0.61 | 0.77 | 0.27 | -0.54 | 0.32 | 0.58 | 0.64 | 0.45 | 0.84 | -0.28 | -0.03 | 0.80 | 1.0 |  |  |  |  |
|  | NO_2_ | 0.05 | 0.15 | 0.05 | 0.30 | 0.41 | 0.33 | 0.83 | 0.04 | 0.49 | 0.84 | 0.72 | -0.52 | 0.64 | 0.48 | 0.56 | 0.53 | 0.64 | -0.15 | 0.00 | 0.32 | 0.33 | 0.58 | 0.68 | -0.54 | 0.20 | 0.51 | 0.58 | 1.0 |  |  |  |
|  | O_3_ | -0.01 | 0.16 | -0.00 | -0.58 | -0.05 | -0.54 | -0.46 | -0.93 | -0.85 | -0.48 | -0.48 | -0.22 | -0.29 | -0.47 | -0.14 | -0.48 | 0.31 | 0.53 | -0.42 | -0.30 | -0.58 | 0.01 | -0.58 | -0.22 | 0.08 | -0.50 | -0.72 | -0.05 | 1.0 |  |  |
|  | PM_10_ | -0.08 | -0.03 | -0.09 | -0.09 | 0.26 | -0.01 | 0.38 | 0.17 | 0.33 | 0.45 | 0.17 | 0.06 | 0.54 | 0.13 | 0.40 | 0.30 | 0.49 | 0.56 | -0.02 | 0.11 | 0.05 | 0.09 | -0.08 | 0.07 | 0.36 | 0.30 | 0.00 | 0.48 | 0.43 | 1.0 |  |
|  | PM_2.5_ | 0.09 | 0.19 | 0.10 | 0.29 | 0.29 | 0.31 | 0.60 | -0.10 | 0.27 | 0.63 | 0.36 | -0.34 | 0.62 | 0.31 | 0.60 | 0.54 | 0.75 | 0.23 | 0.11 | 0.49 | 0.10 | 0.27 | 0.25 | -0.32 | 0.21 | 0.47 | 0.53 | 0.62 | -0.10 | 0.72 | 1.0 |

^a^ Unseasonalized LUR model estimates.

^b^ Seasonalized LUR model estimates.

^c^ NAPH = naphthalene; BAP= benzo(a)pyrene; BGP= benzo(g,h,i)perylene; BENZ= benzene; AN= ammonium nitrate, AS= ammonium sulfate; BB=biomass burning; D=diesel; G=gasoline; GL=geological; MC=meat cooking; RO=residual oil; SS=sea salt.

^d^ Correlations for TSP V were similar to those presented for PM_2.5_ V.

^e^ Correlations for NO_x_ were similar to those presented for NO.

## Table S-5. Varimax rotation, principal component factors for entire pregnancy air pollution averages.^a^

| **Variable** | **Factor Loadings** | | | | | **Communality**  **(h_i_^2^)** | **Specificity**  **(u_i_^2^)** |
| --- | --- | --- | --- | --- | --- | --- | --- |
|  | **F1** | **F2** | **F3** | **F4** | **F5** |  |  |
| **Factor I** |  |  |  |  |  |  |  |
| PM_10_ OC | **0.95** | 0.04 | -0.11 | 0.19 | 0.19 | 0.99 | 0.01 |
| PM_2.5_ EC | **0.95** | 0.12 | -0.13 | 0.19 | 0.08 | 0.97 | 0.03 |
| Diesel PM_2.5_ | **0.93** | -0.25 | -0.10 | 0.20 | 0.02 | 0.99 | 0.01 |
| PM_2.5_ | **0.93** | -0.26 | 0.09 | 0.19 | 0.05 | 0.98 | 0.02 |
| NO_x_ | **0.91** | 0.08 | -0.32 | 0.21 | 0.05 | 0.98 | 0.02 |
| NO_2_ | **0.91** | -0.25 | -0.22 | 0.21 | 0.09 | 0.98 | 0.02 |
| PM_10_ | **0.90** | 0.08 | 0.39 | 0.14 | -0.06 | 0.99 | 0.01 |
| NO | **0.89** | 0.19 | -0.34 | 0.20 | 0.03 | 0.98 | 0.02 |
| PM_2.5_ OC | **0.87** | -0.36 | -0.20 | 0.21 | 0.05 | 0.98 | 0.02 |
| Total PAHs | **0.87** | 0.20 | -0.24 | 0.18 | 0.28 | 0.97 | 0.03 |
| Ammonium nitrate PM_2.5_ | **0.87** | -0.43 | -0.07 | 0.20 | 0.08 | 0.99 | 0.01 |
| Naphthalene | **0.85** | 0.17 | -0.30 | 0.18 | 0.29 | 0.97 | 0.03 |
| Benzene | **0.82** | -0.08 | -0.46 | 0.21 | 0.20 | 0.98 | 0.02 |
| PM_10_ EC | **0.81** | 0.33 | -0.34 | 0.16 | 0.14 | 0.93 | 0.07 |
| Biomass burning PM_2.5_ | **0.80** | 0.25 | -0.42 | 0.15 | -0.20 | 0.95 | 0.05 |
| CO | **0.73** | **0.61** | 0.16 | 0.11 | -0.05 | 0.95 | 0.05 |
| **Factor II** |  |  |  |  |  |  |  |
| Sea salt PM_2.5_ | -0.11 | **0.94** | 0.08 | -0.08 | -0.14 | 0.93 | 0.07 |
| Benzo(a)pyrene | 0.18 | **0.93** | -0.21 | 0.003 | 0.11 | 0.95 | 0.05 |
| O_3_ (10am-6pm) | -0.22 | **-0.92** | 0.24 | -0.03 | 0.12 | 0.97 | 0.03 |
| Gasoline PM_2.5_ | 0.17 | **0.90** | 0.34 | -0.01 | 0.07 | 0.96 | 0.04 |
| PM_2.5_ vanadium | -0.43 | **0.76** | 0.43 | -0.15 | -0.15 | 0.99 | 0.01 |
| Residual oil PM_2.5_ | -0.43 | **0.76** | 0.39 | -0.16 | -0.22 | 0.98 | 0.02 |
| TSP vanadium | -0.45 | **0.74** | 0.42 | -0.16 | -0.18 | 0.99 | 0.01 |
| Benzo(g,h,i)perylene | **0.63** | **0.67** | -0.30 | 0.11 | 0.20 | 0.98 | 0.02 |
| **Factor III** |  |  |  |  |  |  |  |
| Ammonium sulfate PM_2.5_ | -0.30 | 0.24 | **0.87** | -0.13 | -0.17 | 0.96 | 0.04 |
| Meat cooking PM_2.5_ | **0.60** | -0.12 | **-0.69** | 0.19 | 0.21 | 0.93 | 0.07 |
| **Factor IV** |  |  |  |  |  |  |  |
| NO_x_ LUR_U^b^ | 0.09 | -0.32 | -0.06 | **0.93** | 0.05 | 0.97 | 0.03 |
| NO LUR_U^b^ | 0.06 | -0.28 | -0.04 | **0.90** | 0.04 | 0.89 | 0.11 |
| NO_2_ LUR_S^c^ | 0.32 | 0.15 | -0.02 | **0.87** | 0.02 | 0.88 | 0.12 |
| NO_x_ LUR_S^c^ | 0.38 | 0.34 | -0.11 | **0.84** | 0.02 | 0.98 | 0.02 |
| NO LUR_S^c^ | 0.42 | 0.27 | -0.15 | **0.80** | 0.02 | 0.91 | 0.09 |
| NO_2_ LUR_U^b^ | 0.27 | -0.52 | -0.11 | **0.75** | 0.10 | 0.93 | 0.07 |
| **Factor V** |  |  |  |  |  |  |  |
| Geological PM_2.5_ | 0.29 | -0.23 | -0.25 | 0.10 | **0.87** | 0.97 | 0.03 |
| Variance explained | 14.45 | 7.53 | 3.36 | 5.03 | 1.38 | 31.8 | 1.2 |
| Percentage of total variance | 43.8 | 22.8 | 10.2 | 15.3 | 4.2 | 96.2 | 3.8 |

^a^ Based on 7,498 subjects with non-missing values for entire pregnancy averages for all exposure metrics.

^b^ Unseasonalized LUR model estimates.

^c^ Seasonalized LUR model estimates.

## Table S-6. Associations between IQR and unit increases in entire pregnancy average air pollution exposures and preterm birth.

| **Exposure Metric** | **IQR** | **Crude** | | **Adjusted^a^** | | **Unit Scaling** | **Crude** | **Adjusted^a^** |
| --- | --- | --- | --- | --- | --- | --- | --- | --- |
|  |  | **N (cases, controls)** | **OR (95% CI)** | **N (cases, controls)** | **OR (95% CI)** |  | **OR (95% CI)** | **OR (95% CI)** |
| NO LUR_U^b^ | 7.5 ppb | 10265, 102650 | 1.04 (1.02-1.06) | 10134, 100467 | 1.03 (1.01-1.05) | 10 ppb | 1.06 (1.03-1.08) | 1.04 (1.02-1.07) |
| NO LUR_S^c^ | 14.5 ppb | 7838, 60115 | 1.01 (0.98-1.04) | 7745, 58874 | 0.99 (0.96-1.02) | 10 ppb | 1.01 (0.98-1.03) | 1.00 (0.97-1.02) |
| NO_2_ LUR_U | 4.2 ppb | 10265, 102650 | 1.04 (1.02-1.07) | 10134, 100467 | 1.04 (1.02-1.07) | 10 ppb | 1.10 (1.04-1.16) | 1.10 (1.04-1.17) |
| NO_2_ LUR_S | 5.6 ppb | 7838, 60115 | 0.96 (0.93-0.99) | 7745, 58874 | 0.97 (0.94-1.00) | 10 ppb | 0.93 (0.88-0.99) | 0.95 (0.90-1.00) |
| NO_x_ LUR_U | 11.6 ppb | 10265, 102650 | 1.04 (1.02-1.06) | 10134, 100467 | 1.03 (1.01-1.05) | 10 ppb | 1.04 (1.02-1.06) | 1.03 (1.01-1.05) |
| NO_x_ LUR_S | 19.8 ppb | 7838, 60115 | 0.99 (0.96-1.02) | 7745, 58874 | 0.98 (0.95-1.01) | 10 ppb | 1.00 (0.98-1.01) | 0.99 (0.97-1.01) |
| Naphthalene | 52.2 ng/m^3^ | 1092, 1914 | 1.28 (1.14-1.44) | 1068, 1859 | 1.29 (1.14-1.45) | 50 ng/m^3^ | 1.27 (1.14-1.42) | 1.27 (1.13-1.43) |
| Benzo(a)pyrene | 0.06 ng/m^3^ | 1092, 1914 | 1.07 (0.97-1.18) | 1068, 1859 | 1.13 (1.02-1.25) | 0.1 ng/m^3^ | 1.13 (0.96-1.34) | 1.24 (1.04-1.48) |
| Benzo(g,h,i)perylene | 0.14 ng/m^3^ | 1092, 1914 | 1.28 (1.12-1.45) | 1068, 1859 | 1.34 (1.17-1.52) | 0.1 ng/m^3^ | 1.20 (1.09-1.31) | 1.24 (1.12-1.36) |
| Total PAHs | 59.3 ng/m^3^ | 1092, 1914 | 1.30 (1.15-1.46) | 1068, 1859 | 1.30 (1.15-1.47) | 50 ng/m^3^ | 1.25 (1.13-1.38) | 1.25 (1.13-1.39) |
| Benzene^d^ | 0.20 ppb | 5856, 31564 | 1.10 (1.07-1.14) | 5787, 30922 | 1.09 (1.06-1.13) | 0.1 ppb | 1.05 (1.03-1.07) | 1.05 (1.03-1.07) |
| PM_10_ OC | 0.99 µg/m^3^ | 6157, 34743 | 1.13 (1.09-1.17) | 6083, 34040 | 1.14 (1.10-1.19) | 1 µg/m^3^ | 1.13 (1.09-1.17) | 1.15 (1.10-1.19) |
| PM_10_ EC | 0.55 µg/m^3^ | 6157, 34743 | 1.05 (1.01-1.09) | 6083, 34040 | 1.06 (1.01-1.10) | 1 µg/m^3^ | 1.09 (1.01-1.17) | 1.10 (1.03-1.19) |
| TSP vanadium | 5.1 ng/m^3^ | 6163, 34831 | 0.96 (0.94-0.99) | 6090, 34137 | 0.95 (0.93-0.98) | 5 ng/m^3^ | 0.96 (0.94-0.99) | 0.95 (0.93-0.98) |
| PM_25_ OC | 1.3 µg/m^3^ | 6146, 34643 | 1.13 (1.09-1.17) | 6072, 33940 | 1.13 (1.09-1.17) | 0.5 µg/m^3^ | 1.05 (1.03-1.06) | 1.05 (1.03-1.06) |
| PM_25_ EC | 0.55 µg/m^3^ | 6146, 34643 | 1.13 (1.09-1.18) | 6072, 33940 | 1.12 (1.08-1.17) | 0.5 µg/m^3^ | 1.12 (1.08-1.16) | 1.11 (1.07-1.15) |
| PM_2.5_ vanadium | 3.5 ng/m^3^ | 6146, 34671 | 0.96 (0.94-0.99) | 6072, 33968 | 0.95 (0.93-0.98) | 5 ng/m^3^ | 0.95 (0.91-0.98) | 0.94 (0.90-0.97) |
| Ammonium nitrate PM_2.5_ | 1.8 µg/m^3^ | 6760, 41618 | 1.18 (1.13-1.24) | 6673, 40737 | 1.21 (1.16-1.27) | 1 µg/m^3^ | 1.10 (1.07-1.12) | 1.11 (1.08-1.14) |
| Ammonium sulfate PM_2.5_ | 1.8 µg/m^3^ | 6760, 41618 | 1.02 (0.99-1.06) | 6673, 40737 | 1.00 (0.97-1.04) | 1 µg/m^3^ | 1.01 (0.99-1.03) | 1.00 (0.98-1.02) |
| Biomass burning PM_2.5_ | 0.15 µg/m^3^ | 6742, 41351 | 1.09 (1.05-1.13) | 6655, 40476 | 1.12 (1.08-1.16) | 0.1 µg/m^3^ | 1.06 (1.03-1.08) | 1.08 (1.05-1.11) |
| Diesel PM_2.5_ | 0.99 µg/m^3^ | 6751, 41469 | 1.10 (1.06-1.14) | 6664, 40592 | 1.11 (1.07-1.15) | 0.5 µg/m^3^ | 1.05 (1.03-1.07) | 1.05 (1.03-1.07) |
| Gasoline PM_2.5_ | 0.71 µg/m^3^ | 4657, 18523 | 0.98 (0.93-1.03) | 4605, 18204 | 0.95 (0.90-1.00) | 0.5 µg/m^3^ | 0.99 (0.95-1.02) | 0.97 (0.93-1.00) |
| Geological PM_2.5_ | 0.64 µg/m^3^ | 6542, 38138 | 0.97 (0.94-1.01) | 6462, 37364 | 1.00 (0.96-1.04) | 0.5 µg/m^3^ | 0.98 (0.95-1.01) | 1.00 (0.97-1.03) |
| Meat cooking PM_2.5_ | 0.69 µg/m^3^ | 5443, 26557 | 1.04 (1.00-1.08) | 5374, 26029 | 1.02 (0.98-1.06) | 0.5 µg/m^3^ | 1.03 (1.00-1.06) | 1.01 (0.98-1.04) |
| Residual Oil PM_2.5_ | 0.23 µg/m^3^ | 6760, 41618 | 0.97 (0.95-0.99) | 6673, 40737 | 0.96 (0.94-0.98) | 0.1 µg/m^3^ | 0.99 (0.98-1.00) | 0.98 (0.97-0.99) |
| Sea Salt PM_2.5_ | 0.45 µg/m^3^ | 6760, 41618 | 1.09 (1.05-1.13) | 6673, 40737 | 1.07 (1.03-1.11) | 0.5 µg/m^3^ | 1.10 (1.06-1.15) | 1.08 (1.03-1.13) |
| CO | 0.38 ppm | 7957, 61958 | 0.98 (0.95-1.02) | 7862, 60685 | 0.96 (0.92-0.99) | 0.5 ppm | 0.98 (0.94-1.02) | 0.94 (0.90-0.98) |
| NO | 19.8 ppb | 7936, 61493 | 1.00 (0.97-1.03) | 7843, 60240 | 0.99 (0.95-1.02) | 10 ppb | 1.00 (0.98-1.02) | 0.99 (0.98-1.01) |
| NO_2_ | 4.3 ppb | 7936, 61493 | 1.00 (0.97-1.03) | 7843, 60240 | 1.01 (0.97-1.04) | 10 ppb | 1.00 (0.93-1.07) | 1.01 (0.94-1.09) |
| NO_x_ | 22.8 ppb | 7936, 61493 | 1.00 (0.97-1.03) | 7843, 60240 | 0.99 (0.96-1.03) | 10 ppb | 1.00 (0.99-1.02) | 1.00 (0.98-1.01) |
| O_3_ (10am-6pm) | 8.2 ppb | 7957, 61958 | 0.99 (0.96-1.02) | 7862, 60685 | 1.02 (0.98-1.05) | 10 ppb | 0.99 (0.95-1.03) | 1.02 (0.98-1.07) |
| PM_10_ | 5.8 µg/m^3^ | 4869, 24002 | 0.97 (0.93-1.02) | 4807, 23476 | 0.99 (0.95-1.04) | 10 µg/m^3^ | 0.96 (0.88-1.04) | 0.99 (0.91-1.08) |
| PM_2.5_ | 2.6 µg/m^3^ | 8399, 69099 | 0.91 (0.88-0.94) | 8306, 67740 | 0.91 (0.88-0.94) | 5 µg/m^3^ | 0.83 (0.79-0.88) | 0.84 (0.79-0.89) |

^a^ Adjusted for maternal age, race/ethnicity and education, and parity.

^b^ Unseasonalized LUR model estimates.

^c^ Seasonalized LUR model estimates.

**REFERENCES**

Beckerman B, Jerrett M, Brook JR, Verma DK, Araine MA, Finkelstein MM. 2008. Correlation of nitrogen dioxide with other traffic pollutants near a major expressway. Atmos Environ 42:275–290.

Cho A, Sioutas C, Miguel AH, Kumagai Y, Schmitz D, Singh M, et al. 2005. Redox activity of airborne particulate matter at different sites in the Los Angeles Basin. Environ Res 99:40-47.

Perera FP, Rauh V, Whyatt RM, Tsai WY, Bernert J, Tu Y, et al. 2004. Molecular evidence of an interaction between prenatal environmental exposures and birth outcomes in a multiethnic population. Environ Health Perspect 112:626-630.

SCAQMD (South Coast Air Quality Management District). 2008. Multiple Air Toxics Exposure Study in the South Coast Air Basin: MATES III Final Report. Diamond Bar, CA: South Coast Air Quality Management District.
